# Patient-Centered Outcomes in Non-Melanoma Skin Cancer Management: A Comprehensive Review

**DOI:** 10.1177/12034754251375044

**Published:** 2025-09-11

**Authors:** Megha Udupa, David Roberge, Han Zhang Huang, Kevin Pehr

**Affiliations:** 1 Faculty of Medicine and Health Sciences, McGill University, Montreal, QC, Canada; 2 Département de Radiologie, Radio-Oncologie, et Médecine Nucléaire, Université de Montréal, Montreal, QC, Canada; 3 Department of Oncology, McGill University, Montreal, QC, Canada; 4 Division of Dermatology, Department of Medicine, McGill University, Montreal, QC, Canada; 5 Lady Davis Institute for Medical Research, Montreal, QC, Canada

**Keywords:** non-melanoma skin cancer (NMSC), patient satisfaction, quality of life, basal cell carcinoma (BCC), squamous cell carcinoma (SCC)

## Abstract

Non-melanoma skin cancer (NMSC) is the most prevalent type of cancer worldwide, with a significantly rising incidence. While postoperative patient satisfaction and quality of life (QoL) are key metrics in cancer care, they are understudied with regard to NMSC care. This review aimed to summarize the existing data investigating the QoL outcomes after treatment of NMSC and the determinants of patient satisfaction in NMSC management. PubMed, Embase, Ovid MEDLINE, CINAHL, and Cochrane Library databases were searched up to December 1, 2023. Twenty eligible studies were identified, with 7 examining patient satisfaction, 12 examining QoL, and 1 looking at both. The studies used various tools, with the Patient Satisfaction Questionnaire being the most common for assessing patient satisfaction, and the Skin Cancer Index for QoL. Many factors (some controllable, others non-controllable) were found to influence postoperative patient satisfaction, such as preoperative QoL and interpersonal manners of the providers. QoL outcomes were often but not always linked with patient satisfaction and influenced by several factors, such as age, pretreatment mental health, and tumor localization. Clinicians should consider patient perspectives when determining the effectiveness of interventions for NMSC patients. Understanding the factors that influence patient satisfaction and QoL is crucial in delivering comprehensive patient care.

## Introduction

Postoperative patient-centered outcomes are important metrics in cancer care, with patient satisfaction and quality of life (QoL) after treatment being 2 primary examples.^1^ Patient satisfaction refers to how patients view the care they receive for their condition, including not only the treatment itself but also interactions with healthcare personnel and facilities.^2^ QoL encompasses physical, mental, and social health, and considers how the disease and its treatment affect patients’ daily lives.^3,4^

Non-melanoma skin cancer (NMSC) is the most prevalent type of cancer worldwide, comprising primarily basal cell carcinoma (BCC) and squamous cell carcinoma (SCC).^5-7^ The number of new cases attributable to NMSC worldwide was reported to be close to 6.4 million in 2019.^8^ However, the total incidence cannot be reliably estimated as some cancer registries do not include BCC or any NMSC.^9^ The available evidence indicates that the numbers are vast: 77.9 cases/100,000 for females and 122.1 cases/100,000 for males globally.^9^ In Canada, it is estimated that about 80,000 Canadians are diagnosed annually with NMSC, accounting for about 28% of all new cancer cases in Canada.^10^ An upward trend is anticipated to persist.^7,11^

To date, despite the high prevalence of NMSC, little is known about the effects of treatment on QoL and the determinants of patient satisfaction in the management of NMSC.^12-14^ Although NMSC may not always be fatal, its impact is significant; patients are often left with significant functional or psychosocial issues secondary to disfigurement from disease progression or treatment.^7,15^ Numerous treatment modalities exist for NMSC, including classic “cold steel” surgical techniques [excision, Mohs micrographic surgery (MMS)], other destructive modalities [electrodesiccation and curettage (ED&C), cryotherapy, laser], topical chemotherapies, and radiation.^5,6^ Patient satisfaction and QoL therefore emerge as crucial outcome measures in assessing the effectiveness and acceptability of these healthcare interventions, especially in today’s era of emphasis on shared decision-making.^5^ Our review provides a comprehensive synthesis of the current evidence on patient satisfaction and QoL after treatment of NMSC, addressing the factors influencing these outcomes. In this study, we focus on traditional treatment modalities, such as MMS, ED&C, and excision, as advanced therapies remain limited, and our aim is to provide the most generalizable information.

## Materials and Methods

The Preferred Reporting Items for Systematic Reviews and Meta-analyses guidelines were used to guide methodology. A literature search was performed independently by 2 searchers and reviewers (M.U. and H.Z.H.) on the following databases: PubMed, Embase, Ovid MEDLINE, CINAHL, and Cochrane Library until December 1, 2023. Any discrepancy was resolved through consultation with a third reviewer (K.P.). Search terms used included “non-melanoma skin cancer,” “basal cell carcinoma,” “squamous cell carcinoma,” “skin neoplasms,” “patient satisfaction,” and “quality of life.” Papers included were randomized controlled trials, prospective cohort studies, cross-sectional studies, and relevant research letters. No restrictions on geography or date of publication were applied. Exclusion criteria were: if they were case reports/series, conference abstracts, or literature reviews; if the free full text was not accessible; or if they were in a language other than English or French. In fact, no article was excluded for language, and all full texts were retrievable. Studies were considered eligible if they: solely focused on NMSC; assessed patient satisfaction and/or QoL in the context of NMSC treatment; and used at least 1 validated patient-reported outcome instrument. Additional papers were manually retrieved by searching through the reference list of relevant papers. Two independent reviewers (M.U. and H.Z.H.) screened abstracts and full texts against the inclusion criteria. The following information was extracted (M.U. and H.Z.H.) for each article: study characteristics (authors, year, design), patient numbers, interventions, tools used to measure patient satisfaction or QoL, and patient-reported outcomes.

## Results

### Characteristics of Included Studies

The initial search yielded 3322 articles (Figure S1). After application of inclusion and exclusion criteria, 20 papers were included in the present review. Of the included papers, 1 study (5%) was a randomized controlled trial, 14 (70%) used a prospective study design, and 5 (25%) were cross-sectional studies. Seven (35%) examined patient satisfaction, 12 (60%) examined QoL, and 1 (5%) looked at both.

### Patient-Reported Outcome Instruments

Different validated patient-reported outcome instruments were used, including the Medical Outcomes Study Short-Form (SF) Health Survey (12-, 20-, 36-item), Patient and Observer Scar Assessment Scale (POSAS), Dermatology Life Quality Index (DLQI), Skindex, Skin Cancer Index [SCI (12- and 15-item)], Visual Analog Scale (VAS) for pain, Vancouver Scar Scale, Patient Satisfaction Questionnaire (PSQ), FACE-Q Skin Cancer Module, Functional Assessment of Cancer Therapy—General (FACT-G), European Organization for Research and Treatment of Cancer validated PSQ (EORTC-QLQ-SAT32), Hospital Anxiety and Depression Scale (HADS), Importance of Appearance Scale (IAS), UK Sickness Impact Profile, and Lawton Instrumental Activities of Daily Living Scale. Tables S1 and S2 summarize the instruments used and the various parameters assessed in each instrument. Across the 20 studies, some used more than 1 tool to assess patient-reported outcomes. Most commonly, they combined tools for skin-related QoL with a general survey to measure general health status.^11,13,16-19^

Overall, although all instruments are validated, they represent a heterogeneous group. Of the 20 studies, the most used tools were the SCI (n = 6/20, 30%), DLQI (n = 5/20, 25%), PSQ (25%), SF Health Survey (n = 4/20, 20%), and FACE-Q Skin Cancer Module (n = 2/20, 10%). The SCI, although developed and initially published in otolaryngology literature, is specifically geared for NMSC, albeit for MMS of the head and neck. It is a 15-item questionnaire validated in 2004 to evaluate the impact of skin cancer on QoL.^20^ The DLQI is a validated instrument developed for skin diseases, although not specific to skin cancer. It consists of 10 questions grouped into 6 headings (symptoms and feelings, daily activities, leisure, work and school, personal relationships, and treatment) that measure different aspects of health-related QoL.^6^ The PSQ looks beyond the purely aesthetic results, in that it measures patient satisfaction in domains such as technical quality, interpersonal manner of the medical/surgical team, communication, financial aspects, time with clinician, and accessibility.^18,21^ The SF Health Survey, with the most common version being SF-36, is a general tool that assesses the QoL in the domains of physical function, role function, social function, mental health, current health, and pain.^18^ Finally, the FACE-Q includes a number of validated instruments developed at Memorial Sloan Kettering Cancer Center and used to quantify health-related QoL after surgery of facial skin cancers through scales like facial appearance, appraisal of scars, cancer worry, and psychosocial distress.^22,23^

### Predictors of Patient Satisfaction

Table S3 shows the characteristics of the 7 studies that assess patient satisfaction, as well as the 1 that also assesses QoL, for a total of 8 studies.

In a study by Asgari et al,^5^ a large cohort of 834 patients treated by either MMS, excision, or ED&C was examined. Short-term patient satisfaction was measured 1 week after therapy with the 18-item version of the PSQ. To assess pretreatment status, tumor-related QoL was measured with the 16-item version of Skindex, and health status was measured with an adapted version of the Medical Outcomes SF-12 survey, which reported a physical component score and mental component score. To measure long-term patient satisfaction 1 year after therapy, a single global question, “I am completely satisfied with the treatment of my skin problem,” was used, which was derived from the general satisfaction items of the PSQ-18. A global item was used based on the reasoning that after a year, patients are more likely to remember their overall impressions rather than the specific details of their experience. In terms of short-term patient satisfaction, patients were found to be more satisfied with the interpersonal manners of the staff, communication, and financial aspects of their care, compared to the technical quality, time with the clinician, and accessibility of their care. In terms of long-term patient satisfaction, determinants of higher satisfaction included younger age, better preoperative skin-related QoL, better preoperative mental health status, and treatment with MMS.

A prospective cohort study by Thompson et al^14^ including 100 patients, noted that multiple factors were associated with decreased patient satisfaction with MMS for NMSC. The 18-item PSQ was administered at the time of surgery (after at least 1 MMS stage had been excised) and readministered 3 months post-surgery along with 3 scales from the FACE-Q Skin Cancer module (Appraisal of Scars, Cancer Worry, and Satisfaction with Information). In terms of patient satisfaction at the time of surgery, determinants of lower satisfaction included 3 or more MMS stages (*P* = .047)—also noted at 3 months (*P* = .024)—and morning procedures ending after 01:00 PM (*P* = .019). Both are related to the time spent in surgery. Interestingly, early arrival to appointments correlated with increased satisfaction (*P* = .005). In terms of long-term patient satisfaction, a decrease in satisfaction as measured by the PSQ from the time of surgery to 3 months post-surgery was associated with extremity sites [odds ratio (OR) 0.037, 95% confidence interval (CI): 0.002-0.810, *P* = .036], larger preoperative lesion sizes (*P* *=* .012), and larger postoperative defect sizes (*P* *=* .033). Patients with larger preoperative lesion sizes also reported lower Satisfaction with Information scores in the FACE-Q questionnaire at 3 months (*P* *=* .049), which the authors hypothesize may be related to the patient education they received regarding expected wound healing. However, patients undergoing MMS are a minority of NMSC cases; therefore, the results may not be generalizable to all NMSC patients.

On the other hand, another study by Asgari et al,^12^ also focusing on MMS, found that more intraoperative MMS stages, along with better preoperative skin-related QoL (measured with Skindex), were found to significantly increase short-term and long-term patient satisfaction in a sample of 339 patients, with ORs of 7.06 (95% CI: 2.02-24.67) and 5.30 (1.24-22.64), respectively, for more intraoperative MMS stages, and 2.33 (1.01-5.35) and 5.19 (1.66-16.29), respectively, for preoperative skin-related QoL. In addition, patients who were also not bothered by postoperative bleeding (2.25; 1.25-4.05) and who considered themselves involved in the decision-making about treatment choice (3.05; 1.52-6.10) were more likely to be satisfied in the short term. Patients who were married were also more likely to be satisfied in the long term (2.36; 1.10-5.09).

Sasor et al^23^ described 52 patients from the veteran population with facial NMSC who were treated by excision. They were invited to complete a preoperative and postoperative FACE-Q Skin Cancer Module, measured at 1 and 3 months after treatment. The authors found that before and after surgery, veterans were satisfied with their facial appearance, were not bothered by scars, and had low levels of appearance-related psychosocial distress. Patients were also happier with their appearance postoperatively and more satisfied with their scars over time. The mean score regarding cancer worry also decreased after excision. Overall, they were pleased with the outcome and care received. This is an important finding, although the sample was limited to veterans.

Petrosyan et al,^24^ using the EORTC-QLQ-SAT32, sought to compare patient-reported outcomes in 179 patients at their initial consultation, treatment, and follow-up appointments for NMSC of the head and neck. Patients were most satisfied with their treatment appointments (Kruskal-Wallis *P* < .001, *H* = 50.12), and the overall satisfaction score for doctors and nurses was greater than that for service and care organizations (*P* < .001, *H* = 17.85). They suggested that the former could be related to the relief of having their cancer removed and the latter due to the personal nature of the interactions with healthcare providers during the treatment service.

Galles et al^21^ also used the 18-item PSQ to measure satisfaction specifically after treatment with ED&C and compared outcomes with excision and MMS, assessing a total of 717 patients. The PSQ was administered 3 months after treatment. Patients treated with ED&C were less satisfied with the time spent with the clinician (*P* = .06) and the accessibility and convenience of their care (*P* = .03) compared to those treated with excision or MMS. Patients treated with ED&C described worse cosmetic appearance (*P* = .02) and were more often bothered by their cosmetic appearance in the long-term (*P* = .02), which was assessed by global questions rather than a validated instrument.

Postoperative telephone call is a practice used in healthcare to ensure patient comfort, ease anxiety, and triage patients who need closer follow-up in disease management.^25,26^ Vance et al^25^ investigated the effects of postoperative telephone follow-up on patient satisfaction after MMS in a prospective, randomized survey study including 104 patients with NMSC. Patients were randomized to the no-call (control) group or call group. The call group received a postoperative phone call in the evening of the surgery. A 5-point Likert scale was used to assess overall patient satisfaction, and the validated POSAS was used to evaluate scar satisfaction. Patients completed surveys at suture removal between 1 and 3 weeks after MMS and at 3-month follow-up visits. Although patients who received a postoperative phone call reported higher overall satisfaction at short-term and long-term follow-up, the authors did not find the difference to be statistically significant (*P* = .80 and *P* = .51, respectively), perhaps in part due to their study’s relatively small sample size. Scar satisfaction as measured by the POSAS also did not vary statistically between the 2 groups at suture removal (*P* = .31) and at 3 months (*P* *=* .73), although scores trended favorably in the call arm.

In a prospective study conducted by Lee et al,^18^ among 226 NMSC patients undergoing treatment by MMS, specifically for patient satisfaction, the PSQ-18 was administered at the time of surgery but not readministered postoperatively. Instead, a question on willingness to undergo future MMS was posed at 1 month, with 97% responding positively. With the PSQ, they found an overall high satisfaction with the treatment, with a mean score of 4.34/5. Patients were most satisfied with factors related to the interpersonal manner of the staff and were least satisfied with accessibility and convenience, and financial aspects of their care. An increased QoL related to mental health was also associated with higher patient satisfaction. Finally, patients on anticoagulation and who smoked had lower satisfaction, particularly within the technical quality domain of the PSQ, albeit not statistically significant (*P* = .063 and *P* = .085, respectively).

### Determinants of QoL

Table S4 shows the characteristics of the 12 studies that assess QoL, as well as the 1 that also assesses patient satisfaction, for a total of 13 studies.

To evaluate QoL, Rhee et al^20^ surveyed 183 patients with NMSC of the head and neck with the SCI administered within 1 week of surgery and 4 months post-surgery, along with the DLQI. In evaluating interactions with time for the SCI outcomes, patients with lower household income, no previous NMSC, and less complex reconstructions were more likely to have greater QoL after surgery. Younger age (<50 years old) was also a predictor for greater QoL improvement, suggesting that older patients might face more struggles dealing with the effects of NMSC treatment. On the other hand, patients of female sex, those with cancers on the lip, and those who had previous treatment for NMSC demonstrated poorer QoL over time.

In another study by Rhee et al,^11^ where the SF-36 and FACT-G scales were administered at the initial visit, sun-protective behaviors were found to be significantly correlated with higher QoL in 5 of the 8 SF-36 subscales (general health, physical, role physical, social, and vitality) and 2 of the 4 FACT-G subscales (functional and physical). Whether sun-protective habits alter following skin cancer treatment needs to be investigated. A significant correlation was also found between QoL and patients’ coexisting illnesses and medical risk factors (*P* < .001).

Steinbauer et al^3^ assessed 52 patients who were being treated for NMSC with the German version of the DLQI. Seventeen percent of patients reported a very large impairment of their QoL (scores ranging 11-20), with the area symptoms and feelings most affected. They found no correlations with known demographic (such as age and gender) or clinical variables in their study.

Blackford et al^16^ evaluated 44 patients with BCC at baseline, 1 week after treatment, and 3 months after treatment with the DLQI. The authors found little impairment associated with the disease at baseline and post-treatment. Although an increase in the disability scores was seen immediately after treatment, the authors acknowledge that this could presumably be due to the pain after minor surgery and the subsequent disturbed sleep. When comparing treatment methods, excision and cryotherapy were the better-tolerated treatments with mean DLQI scores of 2.0 and 1.5, compared with curettage and cautery (3.9) and excision with flap (4.7), although these differences were not statistically significant.

Radiotis et al^19^ undertook a cross-sectional study of 56 patients with BCC and/or SCC who had received surgery. They employed the HADS to assess psychological distress, the IAS to assess the importance individuals place on 3 domains of body-esteem (general appearance, weight, and others’ evaluation of one’s body and appearance), and the SCI for QoL. They found that patients experiencing higher levels of distress were more likely than patients experiencing low levels of distress to have lower QoL [*t* (54) = 2.22, *P* < .05], particularly in the emotional domain [*t* (54) =2.05, *P* < .05]. The most prevalent NMSC-specific concerns were tumor recurrence and the potential size and conspicuousness of the scar. However, it is important to note that a large proportion had been diagnosed at least 6 months prior to recruitment, and distress may be more elevated during the diagnosis and treatment phases.

Chren et al^13^ used Skindex-16 to compare QoL in patients who received treatment for NMSC with either ED&C, excision, or MMS, with a follow-up of 2 years. QoL improved across all domains (symptoms, emotions, functioning) after excision and MMS (*P* < .05), but not after ED&C. ED&C often leaves scars that are larger than the tumor, which may themselves affect skin-related QoL. Chren et al acknowledge that they did not measure patients’ pretreatment preferences or expectations for different therapies, which can affect patient satisfaction with therapy.

In the same cohort, Chen et al^17^ used Skindex to evaluate predictors of QoL after treatment of NMSC. Better skin-related QoL before treatment was the strongest independent predictor of better QoL after therapy for NMSC. Adjusting for the treatment group, other predictors of better QoL after treatment were better mental health status, less comorbidity, and white race.

Using the DLQI in 255 patients with BCC or SCC, Çetinarslan et al^27^ showed that there was a significant improvement in QoL in both BCC and SCC groups 3 months after surgery when compared with total DLQI scores at baseline (*P* < .001). Gender (*P* *=* .492) and type of skin cancer (*P* = .470) did not affect QoL. However, tumor localization [auricula OR: 6.45 (95% CI: 1.28-37.47)], treatment procedure [flap procedure OR: 7.90 (95% CI: 2.64-23.62) and graft procedure OR: 5.47 (95% CI: 1.60-18.71)], and primary tumor [OR:3.86 (95% CI: 1.01-14.78)] were significant. Worst DLQI scores were seen in the graft group, as graft treatment may have greater associated complications and worse cosmetic results.

In a cross-sectional study conducted by Abedini et al,^6^ 95 patients with NMSC were evaluated with the DLQI. They showed that patients with NMSC faced minimal QoL impairment and that variables associated with impaired QoL were age (Pearson’s correlation coefficient = 0.03), marital status (*P* = .03), and tumor location (*P* *=* .02): younger and single patients and those who had tumors in exposed areas experienced more impairment in their QoL. No significant association was found between DLQI scores and treatment modality.

Sanchez et al^15^ used the SCI to compare QoL outcomes in patients with NMSC who underwent treatment with either MMS or excision. Increased QoL at 2 week follow-up compared to pretreatment was found in patients treated with MMS (*P* < .001), but no significant difference was noted in overall SCI scores in those treated with excision (*P* = .94). The authors recognize that this may be attributed to the decreased sample size in the excision group compared to MMS (n = 30 vs n = 208, respectively).

Lee et al^18^ studied a cohort of 226 patients with MMS and assessed their health-related QoL with the SF-12 and skin-related QoL with the SCI; however, these questionnaires were not readministered postoperatively. Only postoperative complications were measured at 1 month and found to occur in 8.0% of the cohort.

Garcia et al^28^ investigated QoL in patients with cervicofacial NMSC with the Spanish version of the SCI at the time of diagnosis, 1 week, 1, and 6 months after treatment to evaluate the evolution of QoL. Their QoL significantly improved at every time point (*P* < .01). This improvement can be explained by the possibility given to patients of receiving nonsurgical treatment and outpatient surgery options, or treatment requiring a short hospital stay, which could reduce emotional stress.

In another study by García-Montero et al^7^ evaluating the same cohort, sex, education background, marital status, a history of anxiety and/or depression, tumor type, treatment type, and VAS score for the treatment all had a statistically significant impact on the degree of improvement in QoL reported post-treatment.

## Discussion

This review sought to synthesize the current evidence on patient satisfaction and QoL outcomes in NMSC management and care. A total of 20 studies were included: n = 7 researching patient satisfaction, n = 12 researching QoL, and n = 1 researching both parameters. They employed various approaches and instruments to measure the 2 parameters, with the most used tools being PSQ for patient satisfaction (n = 5/20, 25% out of total studies, or 5/8, 63% for just patient satisfaction studies), and SCI for QoL (n = 6/20, 30% out of all studies, or 6/13, 46% for just QoL studies). In addition, some studies used instruments to assess patients’ perception of scars, which is a subcategory of patient satisfaction, since satisfaction with scars post-treatment may be closely linked to overall patient satisfaction.^25^ Despite the high incidence of NMSC, the annual costs, and the numerous treatment modalities, there have been only sporadic attempts to consider what constitutes a “good” treatment from the patients’ perspective.^13^ Since NMSC, especially BCC, are not commonly fatal, we would be remiss in not examining more closely patient-centered factors of satisfaction and QoL.

Our literature search revealed there are currently only 2 systematic reviews related to the present topic: one focuses on the QoL after surgical treatment of BCC,^29^ the other evaluates the characteristics of published patient-reported outcome tools measuring QoL in the dermatologic BCC/SCC population.^1^ To our knowledge, this is the first comprehensive review to examine the determinants of patient satisfaction in NMSC treatment, encompassing a variety of interventions, in addition to being an updated review of the effects of treatment on QoL. Considering the scarce publications on the subject, this is important knowledge for dermatologists when it comes to assessing the best treatment approach for NMSC patients.^3^

Patient satisfaction and QoL are sometimes regarded as one and the same, but different studies use different terms. In addition, patient satisfaction is a purely subjective measure, whereas QoL combines both subjective measures, such as “how do you feel your scar looks,” with more objective measures such as disability.^4^ Therefore, this review has looked at the 2 measures separately. Furthermore, in the context of patient satisfaction, it is crucial to consider QoL outcomes, as these aspects are often interconnected. Studies have shown that increased QoL related to mental health plays a role in predicting higher patient satisfaction.^5,18^ This underscores the importance of addressing factors that can improve pretreatment QoL to enhance overall patient satisfaction outcomes post-treatment.

Looking at the composite of findings, as to what factors were related to patients’ satisfaction or dissatisfaction, as well as what factors are controllable by the treating team and which ones are not, there are notably a few that emerge repeatedly.

Among the controllable factors, the item most often mentioned for a more positive result is interpersonal manners and communication by doctors and other staff.^5,18^ Closely related to this is the amount of time spent with the patient.^21^ For the most part, patients treated by MMS achieve a higher level of satisfaction, at least partly because the time-consuming, staged nature of the technique means that the treating team spends more time with the patient. Asgari et al found that patients who had more stages of MMS had higher satisfaction.^12^ However, Thompson et al found that 3 or more stages of MMS (albeit only a fifth in their statistics) had lower levels of satisfaction, as did those patients whose “morning” surgery finished after 01:00 PM.^14^ Although Asgari’s and Thompson’s studies seem to contradict each other, they can be seen as complementary. With MMS being under local or regional anesthesia, patients are awake the entire time and hence conscious of time passing, both during the surgery and while waiting for the sections to be processed and read. Putting the 2 studies together, patients have increasing satisfaction with time spent, but only up to a certain point (3 sections or 01:00 PM), after which satisfaction decreases.

Among the factors largely beyond the control of the treating team, patients who had a better QoL before the operation showed better outcomes postoperatively,^5,12,17^ as did those who were married.^12^ Negative factors at least partially under the control of the attending physician are chiefly physical appearance, postoperative pain, and postoperative bleeding.^12,21,23^ Appearance is partly dependent on the technique used and the skill of the surgeon, and partly dependent on the size and location of the tumor itself. A major negative factor, which may be outside the physicians’ and nurses’ control, was financial issues.^18^ Even in systems where the direct care is completely covered, there are incidental or indirect costs such as dressings or time lost from work. Patients were also largely dissatisfied with “organizations” (such as hospitals) as opposed to their individual doctors and nurses.^24^

### Limitations and Strengths

A limitation of this review includes heterogeneity of methodology across studies, which prevented statistical analyses of the data. We, therefore, opted for a narrative synthesis approach. The heterogeneity in the instruments used in the studies also made it challenging to compare the scores obtained and to draw definitive conclusions. Furthermore, while some studies addressing patient satisfaction and QoL noted whether an improvement or deterioration was observed postoperatively, they did not delineate the underlying causes of these changes.^13,15,28^ Nonetheless, we deemed it crucial to incorporate these articles into our review. Understanding how changes are quantified post-treatment and how the tools capture these parameters is imperative for a better understanding of the impact on patient satisfaction and QoL. Finally, our review does not address patient-reported outcomes with systemic treatment strategies for advanced BCC or advanced SCC, which remains an important area for future investigation.

## Conclusions

Understanding the factors that influence patient satisfaction and the QoL of individuals undergoing treatment for NMSC is crucial. It enables physicians to actively consider ways to increase patient satisfaction and QoL.

Any future NMSC-assessment instruments should be applicable to various NMSC treatment options such as ED&C, MMS, radiotherapy, and topical dermal agents. It is also imperative to consider the time interval between treatment and questionnaire completion since patient-reported outcomes are contingent on this timing. In doing so, the patient-reported outcome tool will allow the comparison of treatment modalities and provide standards in NMSC care to maximize patient satisfaction.

## Supplemental Material

sj-docx-1-cms-10.1177_12034754251375044 – Supplemental material for Patient-Centered Outcomes in Non-Melanoma Skin Cancer Management: A Comprehensive Review Supplemental material, sj-docx-1-cms-10.1177_12034754251375044 for Patient-Centered Outcomes in Non-Melanoma Skin Cancer Management: A Comprehensive Review by Megha Udupa, David Roberge, Han Zhang Huang and Kevin Pehr in Journal of Cutaneous Medicine and Surgery

sj-docx-2-cms-10.1177_12034754251375044 – Supplemental material for Patient-Centered Outcomes in Non-Melanoma Skin Cancer Management: A Comprehensive Review Supplemental material, sj-docx-2-cms-10.1177_12034754251375044 for Patient-Centered Outcomes in Non-Melanoma Skin Cancer Management: A Comprehensive Review by Megha Udupa, David Roberge, Han Zhang Huang and Kevin Pehr in Journal of Cutaneous Medicine and Surgery
