# Supplementary material for: Patient-Centered Outcomes in Non-Melanoma Skin Cancer Management: A Comprehensive Review
Source: J Cutan Med Surg. 2025 Sep 11;30(2):161–8. doi: 10.1177/12034754251375044 (PMC13076964; doi:10.1177/12034754251375044)
Supplement: sj-docx-1-cms-10.1177_12034754251375044 – Supplemental material for Patient-Centered Outcomes in Non-Melanoma Skin Cancer Management: A Comprehensive Review [file sj-docx-1-cms-10.1177_12034754251375044.docx]

**Supplementary Figure**

**
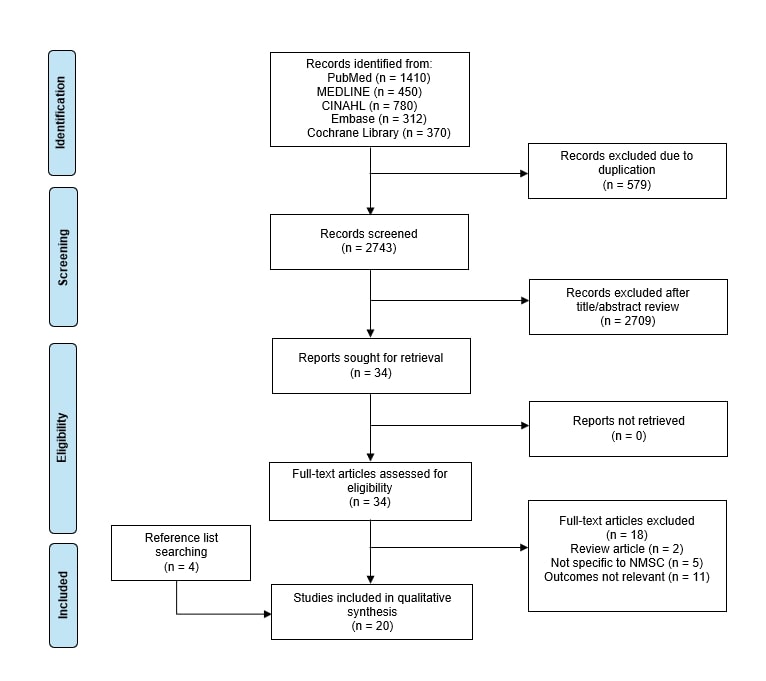
**

**Figure S1.** The preferred reporting items for systematic reviews and meta-analysis (PRISMA) guidelines utilized in the methodology. Adapted from <https://www.prisma-statement.org/>.
