# Supplementary material for: Patient-Centered Outcomes in Non-Melanoma Skin Cancer Management: A Comprehensive Review
Source: J Cutan Med Surg. 2025 Sep 11;30(2):161–8. doi: 10.1177/12034754251375044 (PMC13076964; doi:10.1177/12034754251375044)
Supplement: sj-docx-2-cms-10.1177_12034754251375044 – Supplemental material for Patient-Centered Outcomes in Non-Melanoma Skin Cancer Management: A Comprehensive Review [file sj-docx-2-cms-10.1177_12034754251375044.docx]

**Supplementary Tables**

**Table S1.** Instruments assessing patient satisfaction and their characteristics as used in included studies

| **Instrument** | **Number of items** | **Objective** | **Domains** | **Score Interpretation** |
| --- | --- | --- | --- | --- |
| *General patient satisfaction* | | | | |
| EORTC-QLQ-  SAT32 | 32-item | Measures patient satisfaction with cancer care. | 3 domains: Care received by doctors; Care received by nurses; Service and care organization | Higher scores represent increased satisfaction. |
| FACE-Q Skin  Cancer Module | 56-item (Total for all 7 scales. Each scale can be used independently.) | Measures patient-reported outcomes related to skin cancer treatment and its impact on facial appearance and quality of life, as well as sun protection behavior and adverse effects experienced. | 7 domains: Facial appearance; Appraisal  of scars; Satisfaction with information;  Cancer worry; Psychosocial distress;  Adverse events; Sun protective behavior | Domains may be used separately. All use a 4-point Likert scale. For some scales, higher scores indicate greater satisfaction; for others, higher scores indicate lower satisfaction. |
| POSAS | 2 6-item scales | Measures scar quality by assessing visual, tactile, and sensory characteristics from the observer’s and patient's perspective. | 6 parameters for the observer scale: Vascularization; Pigmentation; Thickness; Relief; Pliability; Surface area.  6 parameters for the patient scale: Pain; Itchiness; Color; Pliability; Thickness; Irregularity. In addition, there is an overall opinion on scar appearance in both scales | Higher scores represent worse perception of scar. |
| PSQ | 18-item | Measures patient satisfaction with medical care. | 7 domains: General satisfaction;  Technical quality; Interpersonal manner; Communication; Financial aspects; Time  spent with clinician; Accessibility and convenience | Higher scores represent increased satisfaction. |
| *Scar satisfaction* | | | | |
| VAS | 1 item | Measures patient perception of pain intensity. | Continuum from “no pain” to “worse pain” | Higher scores represent worse pain. |
| VSS | 4-item | Measures characteristics of the scars produced by treatment. | 4 domains: Pigmentation; Vascularity; Flexibility; Height | Higher scores represent worse healing. |

**Legend.** EORTC-QLQ-SAT32, European Organization for Research and Treatment of Cancer validated Patient Satisfaction Questionnaire; POSAS, Patient and Observer Scar Assessment Scale; PSQ, Patient Satisfaction Questionnaire; VAS, Visual Analog Scale; VSS, Vancouver Scar Scale.

**Table S2.** Instruments assessing QoL outcomes and their characteristics as used in included studies

| **Instrument** | **Number of items** | **Objective** | **Domains** | **Score Interpretation** |
| --- | --- | --- | --- | --- |
| *Skin-related QoL* | | | | |
| DLQI | 10-item | Measures QoL in patients with a skin disease. | 6 domains: Symptoms and feelings; Daily activities; Leisure; Work and school; Personal relationships; Treatment | Higher scores represent worse QoL. |
| SCI | 2 versions: 15-item, 12-item | Measures QoL in patients with NMSC. | 3 domains: Emotional, Social, Appearance | Higher scores represent higher QoL. |
| Skindex | 16-item | Measures effects of skin diseases on QoL. | 3 domains: Symptoms, Emotional effects, Effects on functioning | Higher scores represent worse QoL. |
| *General QoL* | | | | |
| FACT-G | 27-item  (version 4) | Measures health-related QoL in cancer patients. | 4 domains: Physical; Social; Emotional; Functional well-being | Higher scores represent higher QoL. |
| HADS | 14-item | Measures psychological distress. | 2 domains: Depression; Anxiety | Higher scores represent higher psychological distress. |
| IAS | 9-item | Measures importance placed on body-esteem. | 3 domains: General Appearance; Weight; Others’ evaluation of one’s body and appearance. | Higher scores represent greater importance for one’s appearance. |
| Lawton IADL | 8-item | Measures functional status | 8 domains: Using the telephone; Shopping; Preparing food; Housekeeping; Doing laundry; Using transportation; Handling medications; Handling finances. | Higher scores represent higher functional status. |
| Medical Outcomes Study Short-Form Health Survey | 3 versions: 12-item, 20-item, 36-item | Measures general health-related QoL. | 8 domains: physical functioning, vitality, social functioning, general health, bodily pain, physical role, emotional role and mental health.  2 component summary scales: physical and mental component summary scales (PCS and MCS) | Higher scores represent higher QoL. |
| UKSIP | 168-item (grouped in 12 categories) | Measures impact of disease on general health status. | 3 domains: Physical; Psychosocial; Independent | Scores given for each individual scale as well as a weighed single score. Higher scores indicate worse QoL. |

**Legend.** DLQI, Dermatology Life Quality Index; FACT-G, Functional Assessment Cancer Therapy- General; HADS, Hospital Anxiety and Depression Scale; IAS, Importance of Appearance Scale; IADL, Instrumental Activities of Daily Living; NMSC, non-melanoma skin cancer; QoL, quality of life; SCI, Skin Cancer Index; UKSIP, United Kingdom Sickness Impact Profile.

**Table S3.** Characteristics of studies assessing patient satisfaction

| **Authors (Year)** | **Study Type** | **Group Treated** | **Instrument** | **When Instrument was Administered** | **Scores reported*** | **Main predictors of patient satisfaction** |
| --- | --- | --- | --- | --- | --- | --- |
| Asgari et al  (2009) | Prospective cohort study | Patients treated by ED&C, MMS, or excision (n=834) | For short term: PSQ  For long term: Single global question derived from general satisfaction item of PSQ | 1 week after treatment (for short-term)  1 year after treatment (for long-term) | For PSQ:  Short-term (general): 4.19/5 ± 0.86  (mean ± SD)  For global item:  Long-term: 4.08/5 ± 1.08 (mean ± SD) | Short-term: interpersonal manners of the staff, communication, and financial aspects of care predicted higher satisfaction.  Long-term: younger age, better preoperative skin-related QoL, better preoperative mental health status, and MMS predicted higher satisfaction. |
| Asgari et al  (2011) | Prospective cohort study | Patients treated by MMS (n=339) | PSQ,  Single global question derived from general satisfaction item of PSQ | 1 week after treatment (for short-term)  1 year after treatment (for long-term) | For PSQ:  Short-term: 76.1% rated overall satisfaction at 4 or higher on the 5-point scale  For global item:  Long-term: 81.0%  rating their satisfaction at 4 points or higher | Short-term: better preoperative QoL, more MMS stages, no bother from bleeding, and perceived involvement in care predicted higher satisfaction.  Long-term: married, better preoperative skin-related QoL, more MMS stages. |
| Galles et al  (2014) | Prospective cohort study | NMSC patients treated by ED&C, excision or MMS (n= 717) | 18-item PSQ  Global items | 3 months after treatment (short-term)  and one year after treatment (long-term) | For PSQ:  “Time Spent with Clinician”: 3.90 ± 0.8 (ED&C), 4.05 ± 0.8 (excision/MMS)  “Accessibility and Convenience: 3.65 ± 0.9 (ED&C), 3.86 ± 0.8 (excision/MMS)  For global items:  Description of cosmetic appearance: 3.45 ± 1.3 (ED&C), 3.79 ± 1.2 (excision/MMS)  Bother from appearance: 3.06 ± 2.1 (ED&C), 2.37 ± 1.8 (excision/MMS) | ED&C: less satisfied with the time spent with the clinician and the accessibility and convenience of their care compared to those treated with excision or MMS. Also, more  bothered by their cosmetic appearance. |
| Lee et al  (2021) | Prospective cohort study | Patients treated by MMS  (n=226) | PSQ | At treatment (not readministered postoperatively) | 4.34/5 (mean score in the domain of  general satisfaction) | Most satisfied with the interpersonal manners of the staff. Least satisfied with accessibility and convenience and financial aspects of their care |
| Petrosyan et al (2017) | Prospective cohort study | Patients with NMSC of the head and neck  (n=179) | EORTC-QLQ-SAT32 | At initial consultation, treatment and follow-up | **At initial consultation:**  4.38 ± 0.13  (mean summary score ± SD)  **At treatment:** 4.72/5 ± 0.15  **At follow-up:**  4.52 ± 0.23 | Highest scores for satisfaction were seen in the treatment section. The overall satisfaction score for doctors and nurses was greater than that for service and care organization. |
| Sasor et al (2019) | Prospective cohort study | Veterans with facial NMSC treated by excision (n=52) | FACE-Q Skin Cancer Module | Pre-treatment, and 1 month and 6 months after treatment | **Post-op 1 mo** (mean):  Satisfaction with facial appearance= 72  Appraisal of scars= 65  Satisfaction with information (overall)= 100  Cancer worry= 42  Psychosocial distress= 28  Adverse events= 13.0  Sun protective behavior= 14.8  **Post-op 6 mo** (mean):  Satisfaction with facial appearance= 74  Appraisal of scars= 75  Satisfaction with information (overall)= 90  Cancer worry= 47  Psychosocial distress= 20  Adverse events= 13.0  Sun protective behavior= 14.8 | Satisfied with outcome and care received, low levels of psychosocial distress and worry about cancer, and improved sun protection behaviors after treatment. |
| Thompson et al (2023) | Prospective cohort study | Patients treated by MMS (n=100) | PSQ and at 3 months, additional FACE-Q Skin Cancer Module | At treatment and 3 months after treatment | *Only p-values reported*  For PSQ:  **At treatment:**  3 or more MMS stages (P=.0470)  Morning procedures  ending after 1:00 PM  (P = .0190)  Early arrival to appointment (P=.005)  **Post-op 3 mo:**  3 or more MMS stages (P=.0244)  **Change from baseline:**  Surgical sites on the extremities  (P = .0359)  Larger preoperative lesion sizes  (P = .0119)  Larger defect sizes  (P = .0333)  For FACE-Q:  **Post-op 3 mo:**  Satisfaction with Information: Larger preoperative lesions (P= .049) | Short-term: lower satisfaction predicted by 3 or more MMS stages and morning procedures ending after 1:00 PM. Higher satisfaction with earlier arrival to appointment.  Long-term: decrease in satisfaction associated with extremity sites, larger pre-operative lesion sizes, and larger defect sizes, 3 or more MMS stages. |
| Vance et al  (2019) | Randomized, single-blinded, controlled clinical trial | Patients treated by MMS in the phone call arm or control arm (n=104) | General survey for satisfaction and POSAS for scar perception | At suture removal (between 1-3 weeks after treatment) and 3 months after treatment | For general survey:  **At suture removal**: 4.90 vs 4.88 (P= 0.80) in the call vs no-call group, respectively.  **At 3 months**: 4.87 vs 4.76 (P= 0.51)  For POSAS (overall):  **At suture removal**: 3.37 vs 3.81 (P= 0.31) in the call vs no-call group, respectively.  **At 3 months**: 2.73 vs 2.73 (P= 0.73) | Patients in the phone call arm reported higher overall satisfaction at both the suture removal and 3-month surveys compared with  the control “no-call” arm, but not statistically significant.  Scar satisfaction scores did not vary significantly between the two groups. |

**Legend.** ED&C, electrodessication & curettage; EORTC-QLQ-SAT32, European Organization for Research and Treatment of Cancer validated Patient Satisfaction Questionnaire; MMS, Mohs micrographic surgery; NMSC, non-melanoma skin cancer; PSQ, Patient Satisfaction Questionnaire; QoL, quality of life; SD, standard deviation; VAS, Visual Analog Scale. ^*^Overall or total scores reported for the instrument in question. If overall scores not available, subscale scores are provided (unless otherwise indicated).

**Table S4.** Characteristics of studies assessing QoL outcomes

| **Authors (Year)** | **Study Type** | **Group Treated** | **Instrument** | **When Instrument was Administered** | **Scores reported*** | **Main predictors of QoL** |
| --- | --- | --- | --- | --- | --- | --- |
| Abedini et al  (2019) | Cross-sectional study | Patients with BCC or SCC  (n=95) | DLQI | During one year | 4.1 ± 4.25  (mean ± SD) | Poorer QoL: younger age, single patients, and lesions in exposed area. |
| Blackford et al (1996) | Cross-sectional study | Patients with BCC  (n=44) | DLQI for skin-related QoL and UKSIP for general health status | At initial visit, 1  week after treatment and 3 months after treatment | DLQI  **At initial visit:** 5.3 ± 4.1 (overall mean % ± SD)  **At 1 week:** 8.7 ± 9.6  **At 3 months:** 1.3 ± 2.1  UKSIP  **At initial visit:** 0.4 ± 0.8  **At 1 week:** 0.7 ± 1.2  **At 3 months:** 0.13 ± 0.6 | DLQI subcategory “symptoms and feelings” most affected at initial visit and 1-week post-treatment |
| Çetinarslan et al (2020) | Prospective cohort study | Patients with BCC or SCC  (n=255) | DLQI | At baseline and 3 months after treatment | BCC:  **At baseline:** 6.37 ± 6.28 (mean ± SD)  **At 3 months**: 3.96 ± 5.14  SCC:  **At baseline:** 6.35 ± 6.16  **At 3 months:** 4.49 ± 5.24 | QoL affected by tumor localization (worse in auricular and preauricular locations), treatment  procedure (worse in the graft group), tumor type (primary, or recurrent tumor) |
| Chen et al  (2007) | Prospective cohort study | Patients with NMSC (n=633) | Skindex  SF-12 | Pre-treatment and up to 2 years after treatment | No general score reported | Greater QoL (adjusting for treatment): Better pre-treatment skin-related QoL, less comorbidity, better mental health, white race |
| Chren et al  (2007) | Prospective cohort study  *Note: these are the same patients from their prior study above, with different data being presented.* | Patients treated by MMS, ED&C or excision (n=633) | Skindex  SF-12 | Up to 2 years after  treatment | Skindex (mean improvement score, adjusted)  **ED&C:**  Emotional: 5.4  Symptoms: 3.4  Functioning: -1.9  **Excision:**  Emotional: 18.6  Symptoms: 9.7  Functioning: 3.3  **MMS:**  Emotional: 21.7  Symptoms: 10.2  Functioning: 5.0 | Increased QoL across all domains (symptoms, emotions, functioning) after excision and MMS, but not after ED&C. |
| García-Montero et al (2021) | Cross-sectional study of patients followed over time. | Patients with cervicofacial NMSC  (n=220) | 12-SCI | Pre-surgery and post-surgery | **Pre-surgery:** 54.1 ± 21.9  (mean total ± SD)  **Post-surgery**: 61 ± 19.2 | Sex, education background, marital status, a history of anxiety and/or depression,  tumor type, treatment type and VAS score  all exert a statistically significant influence on the improvement during follow-up. |
| García-Montero et al (2022) | Prospective cohort study^#^ | Patients with cervicofacial NMSC  (n=220) | 12-SCI  VAS  VSS | SCI: At the time of diagnosis and at 1 week, 1 month and 6 months after treatment  VAS: 1 week after treatment  VSS: 1 month after treatment | SCI  **At time of diagnosis:** 54.1 ± 21.9 (mean total ± SD)  **At 1 week:** 58.3 ± 20.2  **At 1 month:** 60.1 ± 18.1  **At 6 months:** 61 ± 19.1  VAS  **At 1 week:** 3.7 ± 3  VSS  **At 1 month:** 3.3 ± 2.3 | At the time of diagnosis  patients undergo the greatest deterioration in their QoL. |
| Lee et al  (2021) | Prospective cohort study | Patients treated by MMS  (n=226) | 15-SCI  SF-20  Lawton IADL | At treatment (not readministered post-operatively) | SCI  Emotional: 74.5 (mean)  Social: 86.1  Appearance: 78.6  SF-20  Physical Function: 75.4  Role Function: 76.6  Social Function 87.3  Mental Health: 81.8  Current Health: 68.5  Pain: 67.3 | NR |
| Radiotis et al (2014) | Cross-sectional study | Patients with BCC and/or SCC  (n=56) | 15-SCI  HADS  IAS | At clinical visit | 18% screened positive for clinically significant levels of distress.  Combination of social-, emotional-, and appearance- related QoL significantly predicted psychological distress (F [3, 52] = 5.76, p < 0.01, R2 = 0.25) | Patients experiencing higher levels of distress were more likely to have lower QoL, particularly in the emotional domain. |
| Rhee et al  (2009) | Prospective cohort study | Patients with NMSC  of the face or neck  (n=183) | 15-SCI  DLQI | Pre-surgery and 4 months after  treatment | SCI  **Pre-surgery:** 68.3 (mean total)  **Post-surgery:** 77.5  DLQI  **Pre-surgery:** 2.1 (mean total)  **Post-surgery:** 1.9 | Poorer QoL: female, lip involvement, previous NMSC treatment  Greater QoL: young age (<50 years), lower household income, no previous NMSCs, less extensive reconstructions |
| Rhee et al  (2003) | Cross-sectional study | Patients with cervicofacial NMSC  (n=121) | FACT-G  SF-36 | At initial visit (baseline) | FACT-G  Emotional:  20 ± 3.38  (mean ± SD)  Functional: 24 ± 5.02  Physical: 26 ± 2.60  Social: 25 ± 3.84  SF-36  General health: 74 ± 16.86 Mental: 76 ± 19.42  Pain: 81 ± 21.20  Physical: 85 ± 21.46  Role-emotional: 84 ± 30.15  Role-physical: 78 ± 36.08 Social: 86 ± 21.12  Vitality: 65 ± 20.24 | Sun-protective behaviors were associated with better QoL |
| Sanchez et al  (2020) | Prospective cohort study ^†^ | Patients treated by MMS (n = 208) or excision (n =30) | SCI | Pre-surgery and 2 weeks post-surgery | MMS  **Pre-surgery:** 60.46 (mean total)  **Post-surgery:** 62.82  Excision  **Pre-surgery:** 65.13  **Post-surgery:** 65.07 | Increased QoL after MMS but no such improvement in those treated with excision. |
| Steinbauer et al  (2011) | Prospective observational  study | Patients with NMSC  (n = 52) | DLQI | during the duration  of the outpatient or inpatient stay | 4.90 ± 4.80 (mean ± SD)  17% reported a very large impairment of their QoL (scores 11-20) | Impairment was most pronounced in the area of symptoms and feelings. |

**Legend.** BCC, basal cell carcinoma; DLQI, Dermatology Life Quality Index; ED&C, electrodessication & curettage; FACT-G, Functional Assessment of Cancer Therapy – General; HADS, Hospital Anxiety and Depression Scale; IADL, Instrumental Activities of Daily Living; MMS, Mohs micrographic surgery; NMSC, non-melanoma skin cancer; NR, not recorded; QoL, quality of life; SCC, squamous cell carcinoma; SCI, Skin Cancer Index; SD, standard deviation; SF, Short Form Health Survey; UKSIP, United Kingdom Sickness Impact Profile; VAS; Visual Analog Scale; VSS, Vancouver Scar Scale.  ^*^Overall or total scores reported for the instrument in question. If overall scores not available, subscale scores are provided (unless otherwise indicated). ^#^ Same patients from their 2021 study but looking at a different aspect of care.  ^†^Described as a retrospective study, but questionnaires were administered before and after treatment.
